# O2-1 Improving diet and physical activity in older adults living at home: protocol for the ALAPAGE cluster randomized controlled trial

**DOI:** 10.1093/eurpub/ckac094.009

**Published:** 2022-08-29

**Authors:** Agnès Vinet, Aurélie Bocquier, Christophe Dubois, Pierre Verger, Nicole Darmon

**Affiliations:** 1 LaPEC Laboratoire de Pharm-Ecologie Cardiovasculaire, University of Avignon, Avignon, France; 2 ORS PACA, Marseille, Aix Marseille Univ, IRD, AP-HM, SSA, VITROME, Marseille, Marseille, France; 3 UMR MOISA, INRAE, Marseille, France; 4 UMR MOISA, INRAE, Montpellier, France

**Keywords:** older adults, healthy diet, physical activity, co-construction

## Abstract

**Background:**

Adequate nutrition and regular physical activity (PA) are key elements in healthy aging. In France, behavioural interventions promoting healthy eating and PA in older adults consist mainly in collective workshops organised by pension and health insurance funds. After analyzing pre-existing workshops, we designed the co-constructed project ALAPAGE to improve these workshops and assess their impact on diet and PA.

**Methods:**

ALAPAGE is a cluster randomized controlled trial; 60 collective workshops in southeastern France will be randomized in a 2:1 (intervention/control) ratio. We will recruit 900 autonomous older adults (? 60 years) living at home and will make specific efforts to recruit socially isolated and/or economically vulnerable people. In the intervention group, collective workshop period will include 7 sessions (1 session/week): 1 introductory, 4 diet and 2 PA (to teach principles of functional dual-task exercise focused on strength, flexibility and physical functioning). During the following 3-months, participants will be recommended to perform exercises as often as possible and will participate in post-workshop activities. The control group will first participate to other types of workshops and then to a diet and PA workshop (waiting-list design).

**Results:**

The dietary practices (using experimental economics), physical activity (battery of field test and habitual PA), quality of life, and cost-effectiveness will be assessed at the first and last session, and 3 months later.

**Conclusion:**

Results will guide decision-makers to organize actions and their dissemination. Transferability to other regions will be facilitated by the fact that key stakeholders involved in ALAPAGE belong to structured national networks.

